# Twin Screw Granulation: An Investigation of the Effect of Barrel Fill Level

**DOI:** 10.3390/pharmaceutics10020067

**Published:** 2018-06-01

**Authors:** Sushma V. Lute, Ranjit M. Dhenge, Agba D. Salman

**Affiliations:** Department of Chemical and Biological Engineering, University of Sheffield, Mappin Street, Sheffield S1 3JD, UK; sushmalute@gmail.com (S.V.L.); ranjitdhenge@gmail.com (R.M.D.)

**Keywords:** twin screw granulation, continuous, barrel fill, lactose, microcrystalline cellulose, tableting

## Abstract

This paper focuses on investigating the influence of varying barrel fill levels on the mean residence time, granule properties (median size, size distribution, and shape), and tensile strength of tablets. Specific feed load (SFL) (powder feed rate divided by screw speed) and powder feed number (PFN) (i.e., powder mass flow rate divided by the product of screw speed, screw diameter, and the material density in the denominator) were considered as surrogates for the barrel fill level. Two type of powders (lactose and microcrystalline cellulose (MCC)) were granulated separately at varying fill levels at different liquid-to-solid ratios (L/S). It was observed that by controlling the barrel fill level, the granule size, shape, and tablet tensile strength can be maintained at specific L/S. It was also noticed that the mean residence time decreased with increasing fill levels in the case of both lactose and MCC powder. However, it was only found to be related to the change in granule size in case of granulating microcrystalline cellulose at varying fill levels. At very high fill levels, granule size decreased, owing to a limited interaction between MCC powder and liquid at high throughput force and short residence time.

## 1. Introduction

Continuous wet granulation using a co-rotating twin screw granulator (TSG) has now become a technique of choice in the pharmaceutical industry. The screws of TSGs can consist of various types of elements (conveying, kneading, distributive, combing etc.) arranged in a specific configuration around the screw shafts. The type of screw elements and the geometry is specific to the TSG manufacturer. There are limited companies that produce twin screw granulators for pharmaceutical applications—namely, Leistritz Extrusionstechnik GmbH–NANO 16; Thermo Scientific–Pharma 11, 16, and 24 TSG and GEA Pharma Systems–ConsiGma™-1 (standalone TSG) and 25 systems (TSG as part of GEA’s continuous tableting line). Each manufacturer has copyrighted screw elements and shaft geometry, length to diameter ratio (L/D), screw-barrel gap and barrel arrangement (fixed or closed shell, clam shell etc.). Several papers have been published around TSGs manufactured by Thermo Scientific and GEA Pharma Systems. 

From the literature, it is clear that most of the previous studies on twin screw granulation have concentrated on the parametric evaluations, which caused them to develop a generalised understanding of the effects of process and formulation variables on the dependent variables (torque, barrel fill, and residence time) and granule properties. Most of these studies considered a ‘changing one single variable at a time’ (COST) approach (in other words, a univariate approach) (as described by Vercruysse, Córdoba Díaz et al. [1]), which means that either powder feed rate, liquid to solid ratio (L/S), or screw speed was varied at a time during a run or experiment. For instance, some studies concentrated on understanding the effects of varying powder feed rate [2,3,4,5,6,7,8,9]; some on L/S [8,9,10,11], while others focused on binder viscosity [7,10,12,13,14,15,16,17,18], screw speed [2,3,5,14,15,18,19,20], and screw configuration [5,11,12,18,19,21,22]. From these COST-based studies, it is clear that this approach results in the production of granules with varying attributes (e.g., size, shape, structure). It is known from some studies that variables such as powder feed rate and screw speed impact the barrel fill/channel fill that can change the shear and compaction forces experienced by powder mass in TSG, thereby influencing the granule size, density, and structure [3].

Although barrel fill is important in twin screw granulation, only a few studies considered barrel fill as a primary or input variable [5,22,23,24,25]. Dhenge, Washino et al. [7] and Lee, Ingram et al. [15] in their studies estimated dimensionless volumetric fill level (i.e., the ratio of the volume occupied by wet mass of powder to the total available volume of screw channel) to understand the material occupancy inside the granulator. Kohlgrüber [26] described the barrel fill level (in twin screw extrusion) in the form of a dimensionless input variable, where powder mass flow rate is divided by the product of screw speed, screw diameter, and the material density in the denominator. Kolter, Karl et al. [27] described specific feed load (non dimensionless) as mass flow divided by the screw speed (i.e., mass transported per screw revolution). Osorio, Sayin et al. [25] further explored the approach of Kohlgrüber [26] of a dimensionless input parameter (i.e., powder feed number (PFN)) in their attempt to develop scale-up rules for three different size twin screw granulators. They noticed that PFN is not sufficiently applicable in ‘scaling up’ different size granulators, however, it is useful in ‘scaling out’ using the same granulator. The ‘scaling out’ in their study meant increasing the production rate or throughput in a granulator. They used one specific powder in their study. Gorringe, Kee et al. [23] investigated the influence of volumetric channel fill of conveying elements (i.e., total volumetric fraction of conveying element channels filled with powder) on the granule attributes. They did not consider kneading zone in the calculation, assuming that it appears filled with powder mass at most of the process parameters. Furthermore, Gorringe, Kee et al. [23] used two powders in their study, however, they did not compare the formulations using the same screw configuration. Lute, Dhenge et al. [24] and Meier, Moll et al. [22] utilized the approach of Kolter, Karl et al. [27] of SFL for one specific drug formulation as a surrogate parameter for the volumetric fill level. Meier, Moll et al. [22] granulated one specific formulation (with high drug loaded, i.e., 89.8% (*w*/*w*) hydrochlorothiazide) at various SFLs at constant L/S (0.11) and specific screw configurations using a 40D long 16 mm TSG from Thermo Scientific. There is a need for further research on this topic in order to understand the impact of varying fill levels on granule and tablet properties when L/S and formulation or powder are varied. Furthermore, Kumar, Alakarjula et al. [28] also emphasized the need for finding and understanding the balance between the powder feed rate and screw speed in order to control the desired product attributes. 

This study attempts to further the understanding of the influence of two surrogates for the overall barrel fill level or occupancy (i.e., specific feed load (SFL) and powder feed number (PFN)) on the mean residence time, and thereby the granulation performance, of lactose (at varying L/S) and microcrystalline cellulose (MCC) powder using a 25D long 16 mm TSG from Thermo Scientific. The PFN approach was used in this study along with SFL as it allows for comparison between the different powders, unlike SFL.

## 2. Materials and Methods

### 2.1. Materials

#### 2.1.1. Powder

Two types of powders were used in the study: α-lactose monohydrate (Pharmatose 200M) (bulk density- 0.5 g/mL) and microcrystalline cellulose (Avicel PH 101) (bulk density- 0.3 g/mL), supplied by DMV-Fonterra Excipient GmbH and Co. (Goch, Germany) and FMC Biopolymer (Cork, Ireland), respectively. The particle size of the powders (μm) was measured using a Camsizer XT (Retsch Technology, Haan, Germany). The lactose powder had particle size characteristics as follows: d_10_—9.3 μm, d_50_—42.1 μm, and d_90_—110.0 μm, while MCC powder had the following characteristics: d_10_—19.0 μm, d_50_—57.0 μm, and d_90_—135.0 μm. 

#### 2.1.2. Granulator

**Figure 1:**
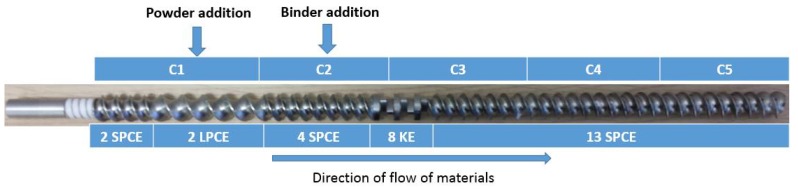
Screw configuration with various compartments. C1-C5: TSG Compartments 1-5; SPCE: Short Pitch Conveying Element; LPCE: Long Pitch Conveying Element; KE: Kneading Element.

A co-rotating twin screw granulator (TSG) (Euro lab 16 TSG (L/D- 25/1), Prism, Thermo Scientific (Thermo Electron GmbH), Karlsruhe, Germany) was used for the granulation experiments. The screw configuration used is shown in Figure 1. The free volume (68.67 cm^3^) for the screw configuration under consideration was determined using Screw Configuration software from Thermo Scientific.

A gravimetric, loss-in-weight twin screw powder feeder (K-PH-CL-24-KT20, K-Tron Soder, Niederlenz, Switzerland) with a pair of co-rotating screws was used to feed powder into the granulator. A peristaltic pump (101U, Watson Marlow, Cornwall, UK) was used to inject granulation liquid (distilled water) into the granulator.

### 2.2. Methods

#### 2.2.1. Granulation

Lactose and MCC powders were granulated separately using a distilled water twin screw granulator. These two powders were selected as they are well known to differ significantly from each other in terms of physical (size, bulk density, drop penetration, solubility, etc.) and mechanical (compressibility) properties. 

The experimental design for the granulation of lactose and MCC powders at varying specific feed load (SFL) (in g) (Equation (1)) and PFN (-) (Equation (2)) is shown in Table 1. Each experiment was repeated three times.

(1) $$\mathrm{SFL}\;=\frac{\mathrm{Powder}\;\mathrm{feed}\;\mathrm{rate}\;}{\mathrm{Screw}\;\mathrm{speed}\;}$$

(2) $$\mathrm{PFN}=\frac{\mathrm{Powder}\;\mathrm{feed}\;\mathrm{rate}}{\mathrm{Powder}\;\mathrm{bulk}\;\mathrm{density}\;\times \;\mathrm{Screw}\;\mathrm{speed}\;\times \;\mathrm{Free}\;\mathrm{space}\;\mathrm{in}\;\mathrm{granulator}}$$

Three different SFLs, 0.041, 0.083, and 0.166, were selected to cover the lower and upper extremes of the granulation conditions while studying the effect on the residence time and granule attributes. Each time, the powder feed rate and speed were adjusted accordingly to maintain the SFL. PFN was further obtained from SFL using powder bulk density and free space in the twin screw granulator. The liquid was also adjusted to maintain the L/S ratio of 0.048, and 0.1 in the case of lactose and 1.0 in the case of MCC. The barrel temperature was maintained at 25 °C (in all barrel compartments). 

#### 2.2.2. Mean Residence Time (MRT) Measurement

The mean residence time was determined using an ultraviolet (UV) spectrophotometer. The blue dye, Patent Blue (Acid Blue 1, Sigma-Aldrich, Saint Louis, MO, USA), was used as a tracer. Approximately 30 mg of dye was introduced into the inlet of the granulator after stabilisation of the operating conditions. The samples at the granulator exit were collected every 5 s until the tracer disappeared in the collected granules. The sample of 2 g of granules from each time point was dissolved separately in 50 mL of distilled water and kept aside for 2 h. The concentration of tracer in the granules was determined using a UV spectrophotometer at a wavelength of λ = 635 nm. The residence time distribution was described by a differential outlet age function [*E*(*t*)] to give the variation of tracer concentration at the exit (Equation (3)) [29]. 

The *E*(*t*) curves represent the variation of the tracer concentration with time at the exit. The area under the curve of the graph of the tracer concentration against the time was normalised by dividing the concentration values by the total area under the curve, giving the following *E*(*t*) values:

(3) $$E\left(t\right)=\frac{C}{\int_{0}^{\infty }Cdt}≅\frac{C}{\sum_{0}^{\infty }C_{i}\Delta t_{i}}$$

where *C* is tracer concentration appearing at the exit at time t. The MRT (*t_m_*) can be calculated using Equation (4).

(4) $$t_{m}=\int_{0}^{\infty }tE\left(t\right)dt≅\;\;\;\frac{\sum t_{i}C_{i}\Delta t_{i}}{\sum C_{i}\Delta t_{i}}$$

#### 2.2.3. Peak Shear Rate

The effect of screw speed was also explained in terms of the peak shear rate exerted on the powder mass during granulation. The peak shear rate of the twin screw granulator was calculated by the following equation (Equation (5)).

(5) $$Peak\;shear\;rate\;=\frac{\pi \times D\times N}{h\times \;60\;}$$

where, *D* = screw diameter (15.6 mm)
   *N* = screw speed (rpm)
   *h* = overflight gap (i.e., gap between screw tip and barrel wall) (0.2 mm).

#### 2.2.4. Analysis of Granules

##### Size and Shape Analysis of Granules

The granules were air dried on a tray at room temperature for 48 h, and then the size and shape of the granules were analysed. The size of the granules was analysed using a Camsizer (free-fall module) (Retsch Technology, Haan, Germany). The shape of the granules was studied using a Keyence Microscope (Milton Keynes, Buckinghamshire, UK).

#### 2.2.5. Tableting

The granules produced at varying SFL–PFN, screw speed, and L/S (0.048, and 0.1 in case of lactose and 1.0 in case MCC) were sieved into different size classes (212–600 µm, 600–1000 µm, 1000–1400 µm, and 212–1400 µm) and compressed in a 12 mm die at 10 kN compression force [1,14] (test speed—1 mm/min) to produce tablets of approximately 405 mg using an Instron testing machine. This was done to study the impact of granule size range on the tablet tensile strength. The granules were not pre-lubricated internally or externally, however, the punch and die of the compression machine were coated with a thin layer of magnesium stearate to minimise sticking and picking. 

##### Analysis of Tablets

The tablets produced were analysed for their dimension (thickness and diameter) and tensile strength. The thickness and diameter were measured using a digital calliper. The tensile strength of the tablet was measured by a diametric compression method, using Zwick/Roell Z 0.5 (Zwick/Roell, Ulm, Germany) instead of an Instron testing machine. This was done because Zwick/Roell provides a more suitable force range when compared with the Instron testing machine. The tablets were compressed diametrically (test speed—1 mm/min) until they fractured. The force-displacement data was recorded. Ten tablets were used for each experimental condition to produce reproducible data. The strength of tablets (σ) was determined by inputting maximum force (*F*), tablet diameter (*D*), and thickness (*T*) into Equation (6) [30,31].

(6) $$\sigma =2\frac{F}{\pi TD}$$

## 3. Results

### 3.1. Lactose Powder

#### 3.1.1. The Effect of Varying SFL–PFN at Different Screw Speed and L/S on Mean Residence Time

**Figure 2:**
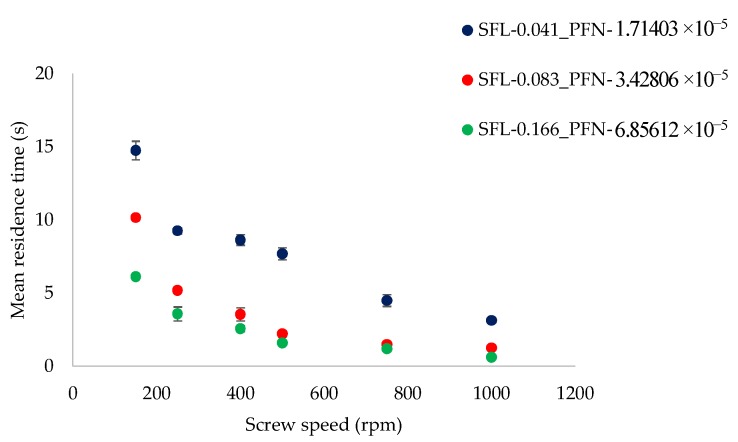
Mean residence time of lactose at different specific feed load (SFL)–powder feed number (PFN) at varying screw speed (L/S 0.048).

**Figure 3:**
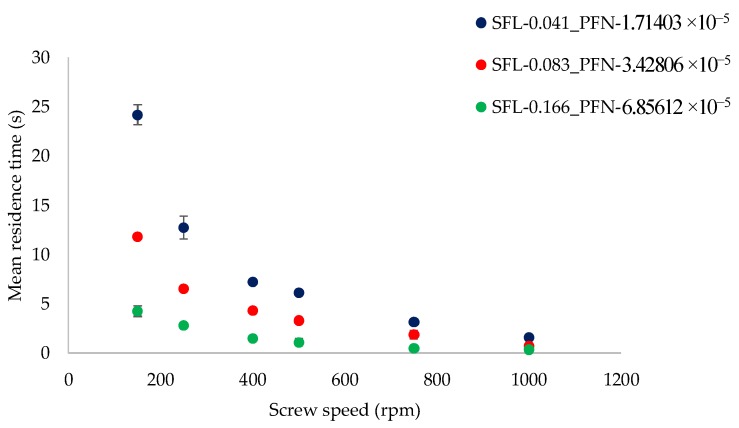
Mean residence time of lactose at different SFL–PFN at varying screw speed (L/S 0.1).

The focus of the present work (on varying SFL–PFN) is on understanding the effect of increasing screw speed at low and high powder feed rates, which is further linked to finding the balance between these two process variables to achieve certain desired product attributes. For this reason, Figure 2 and Figure 3 are plotted as varying screw speed (i.e., peak shear rates) versus the MRT measured while granulating lactose powder at low L/S of 0.048 and high L/S of 0.1, respectively, at different fill levels. 

#### 3.1.2. The Effect of Varying SFL–PFN at Different Screw Speed and L/S on Granule Size

**Figure 4:**
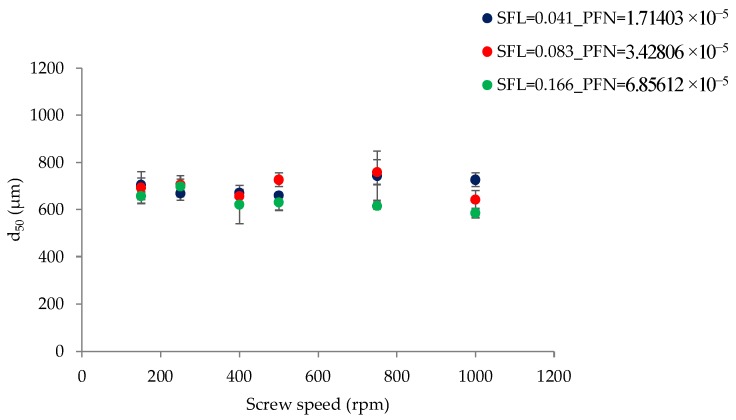
Median granule size of lactose at different SFL–PFN at varying screw speed (L/S 0.048).

Figure 4 shows the effect of varying screw speed (i.e., peak shear rates) on median granule size while granulating lactose powder at low L/S of 0.048 at different fill levels [SFL (0.041–0.166) and PFN (1.71403 × 10^−5^–6.85612 × 10^−5^)].

**Figure 5:**
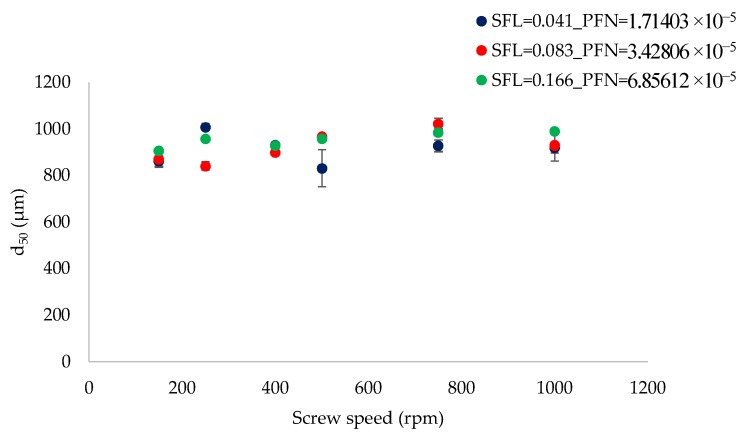
Median granule size of lactose at different SFL–PFN at varying screw speed (L/S 0.1).

Figure 5 shows the effect of varying screw speed on median granule size while granulating lactose powder at relatively higher L/S of 0.1 at different fill levels [SFL (0.041–0.166) and PFN (1.71403 × 10^−5^–6.85612 × 10^−5^)].

**Figure 6:**
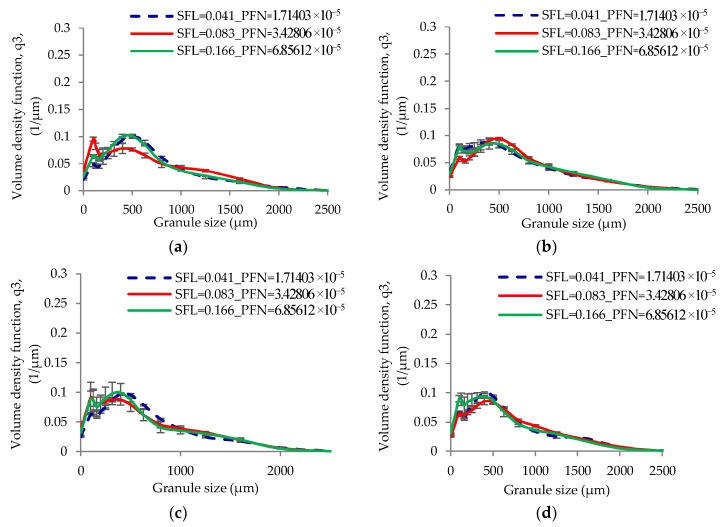

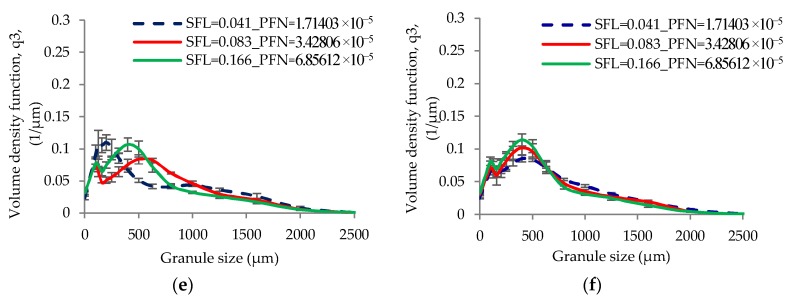
Granule size distribution of lactose (L/S 0.048) at different SFL–PFN at varying screw speed. (**a**) 150 rpm; (**b**) 250 rpm; (**c**) 400 rpm; (**d**) 500 rpm; (**e**) 750 rpm; and (**f**) 1000 rpm.

**Figure 7:**
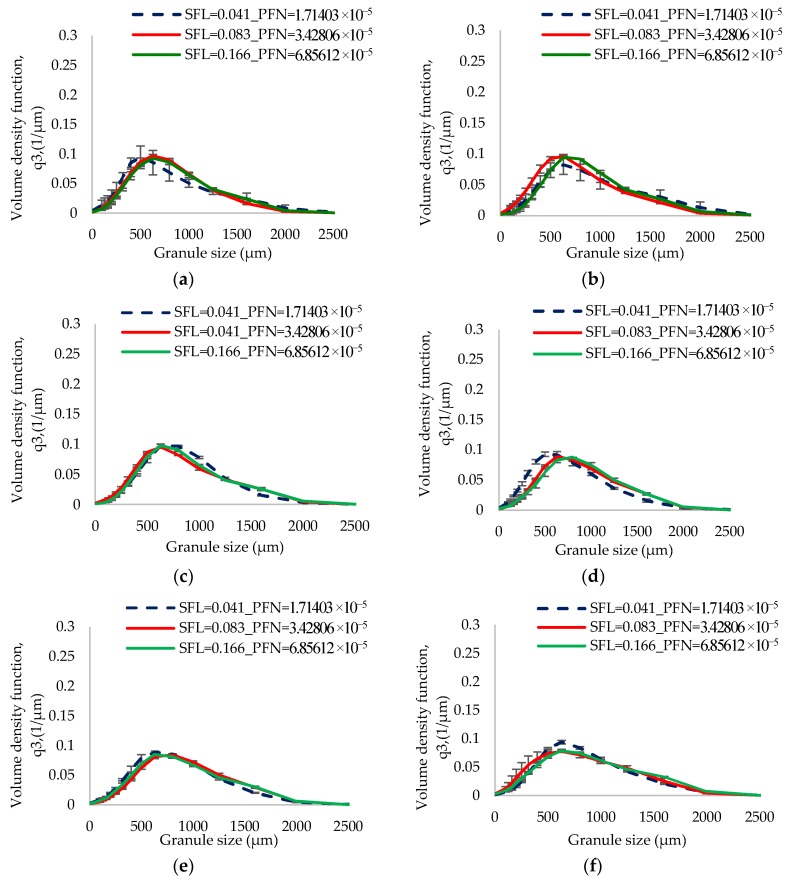
Granule size distribution of lactose (L/S 0.1) at different SFL–PFN at varying screw speed. (**a**) 150 rpm; (**b**) 250 rpm; (**c**) 400 rpm; (**d**) 500 rpm; (**e**) 750 rpm; and (**f**) 1000 rpm.

Figure 6 and Figure 7 show the size distribution of granules produced at varying SFL–PFN at L/S of 0.048 and 0.1, respectively. The size distribution results support the median granule size data obtained at respective L/S.

#### 3.1.3. The Effect of Varying SFL–PFN at Different Screw Speed and L/S on Granule Shape

**Figure 8:**
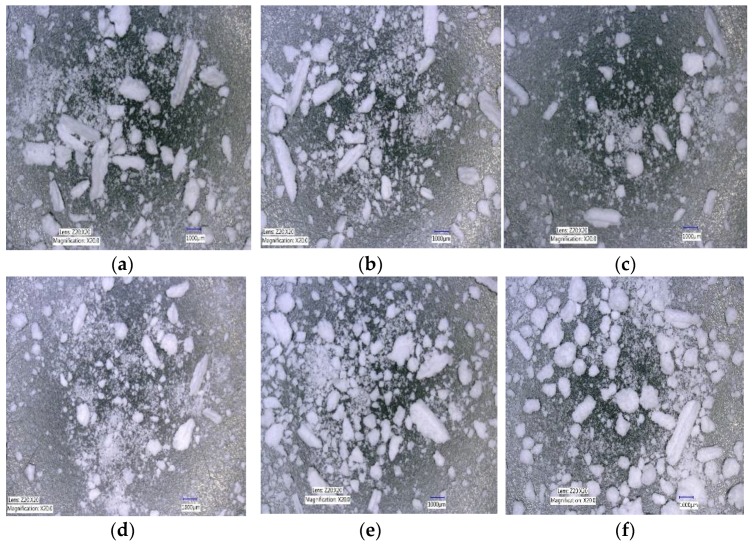
Microscopic images of lactose granules produced at varying screw speed at SFL—0.041 and PFN—1.71403 × 10^−5^ (L/S 0.048). (**a**) 150 rpm; (**b**) 250 rpm; (**c**) 400 rpm; (**d**) 500 rpm; (**e**) 750 rpm; and (**f**) 1000 rpm.

Figure 8a–f shows the shape of granules produced at varying screw speed (i.e., peak shear rates) at low L/S (0.048) and low SFL (0.041) and PFN (1.71403 × 10^−5^).

#### 3.1.4. The Effect of Varying SFL–PFN at Different Screw Speed and L/S on Tablet Tensile Strength

**Figure 9:**
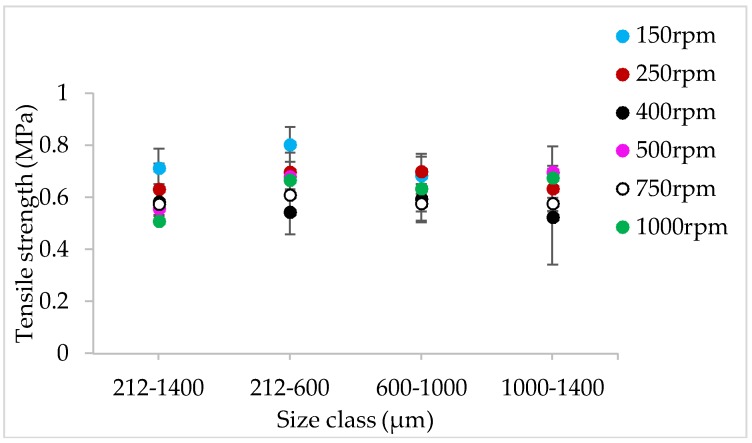
Tensile strength of tablets of lactose granules produced at varying screw speed at SFL—0.041 and PFN—1.71403 × 10^−5^ (L/S 0.048).

**Figure 10:**
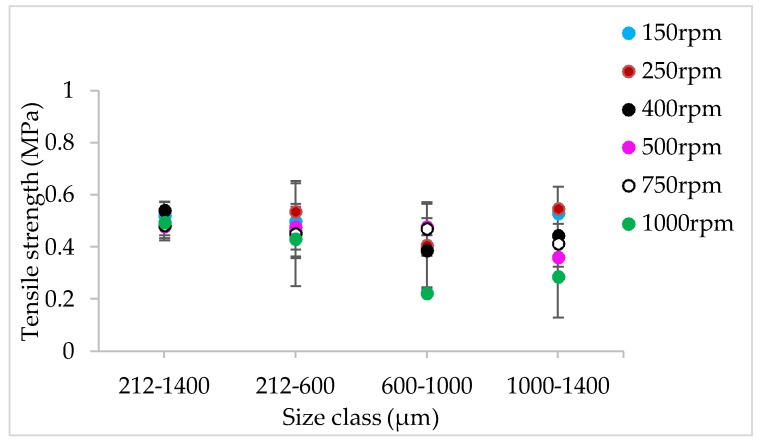
Tensile strength of tablets of lactose granules produced at varying screw speed at SFL—0.041 and PFN—1.71403 × 10^−5^ (L/S 0.1).

**Figure 11:**
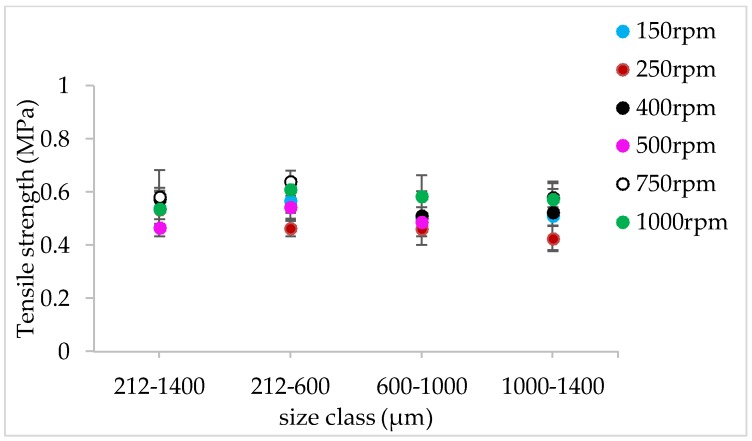
Tensile strength of tablets of lactose granules produced at varying screw speed at SFL—0.083 and PFN—3.42806 × 10^−5^ (L/S 0.048).

**Figure 12:**
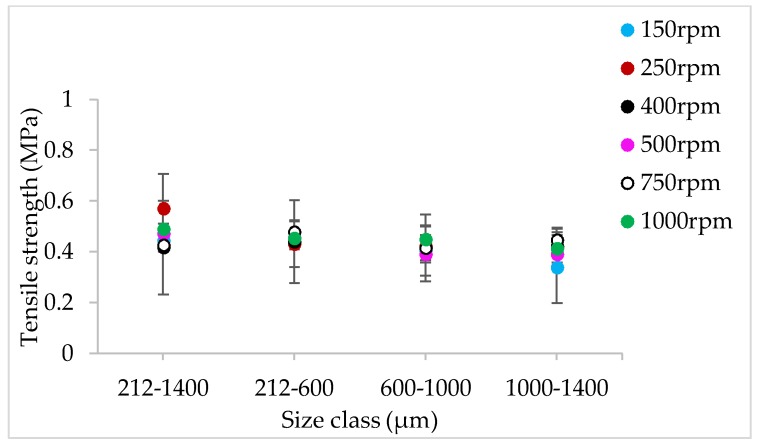
Tensile strength of tablets of lactose granules produced at varying screw speed at SFL—0.083 and PFN—3.42806 × 10^−5^ (L/S 0.1).

**Figure 13:**
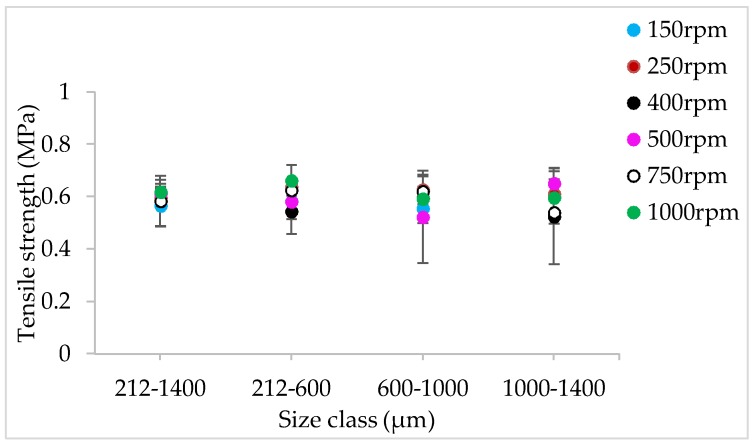
Tensile strength of tablets of lactose granules produced at varying screw speed at SFL—0.166 and PFN—6.85612 × 10^−5^ (L/S 0.048).

**Figure 14:**
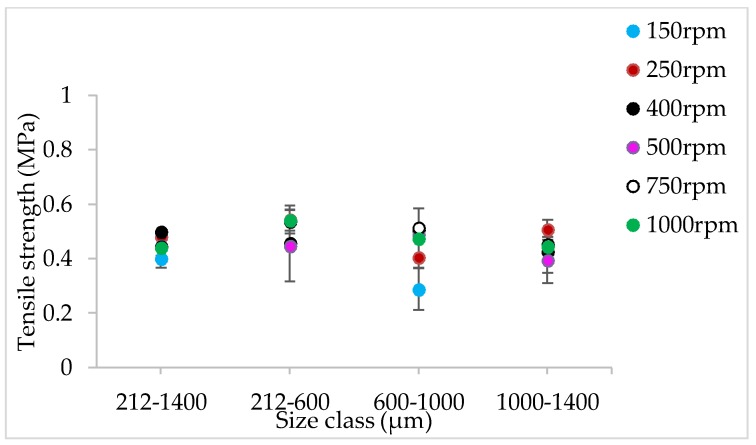
Tensile strength of tablets of lactose granules produced at varying screw speed at SFL—0.166 and PFN—6.85612 × 10^−5^ (L/S 0.1).

Figure 9, Figure 10, Figure 11, Figure 12, Figure 13 and Figure 14 show the tensile strength of tablets of granules (with varying size ranges) produced at varying screw speed, L/S, and SFL–PFN.

### 3.2. Microcrystalline Cellulose (MCC) Powder

#### 3.2.1. The Effect of Varying SFL–PFN at Different Screw Speed on Mean Residence Time

**Figure 15:**
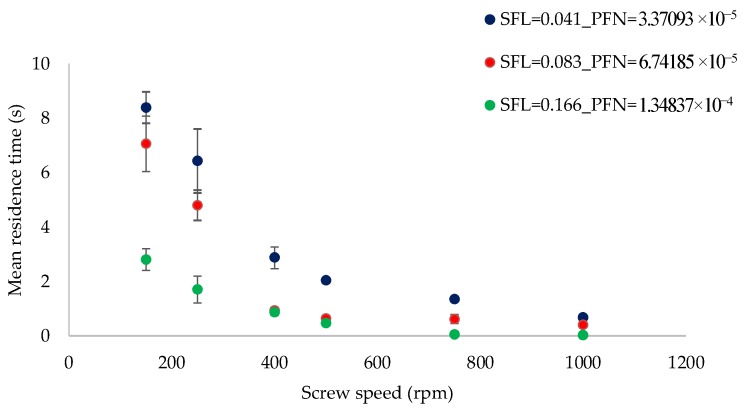
Mean residence time of microcrystalline cellulose (MCC) at different SFL–PFN at varying screw speed (L/S 1).

Figure 15 shows the effect of varying screw speed (i.e., peak shear rate) on MRT measured while granulating MCC powder at constant L/S of 1.0 at different fill levels [SFL (0.041–0.166) and PFN (3.37093 × 10^−5^–1.34837 × 10^−4^)].

#### 3.2.2. The Effect of Varying SFL–PFN at Different Screw Speed on Granule Size

**Figure 16:**
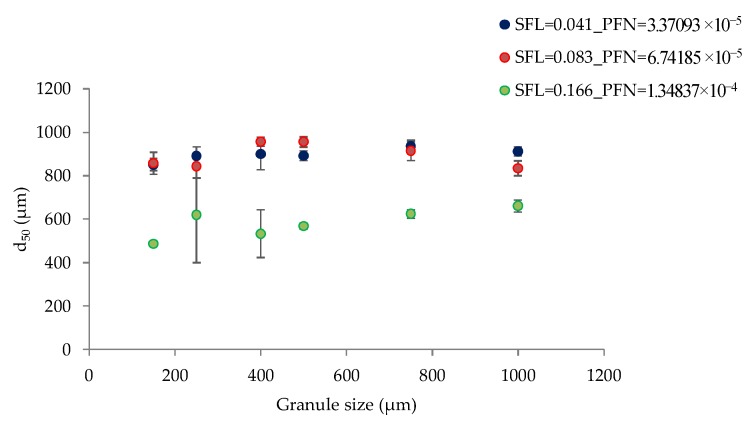
Median granule size of MCC at different SFL–PFN at varying screw speed (L/S 1).

Figure 16 shows the median size of granules of MCC produced at three different fill levels (SFL–PFN) at varying screw speed and constant L/S of 1.

**Figure 17:**
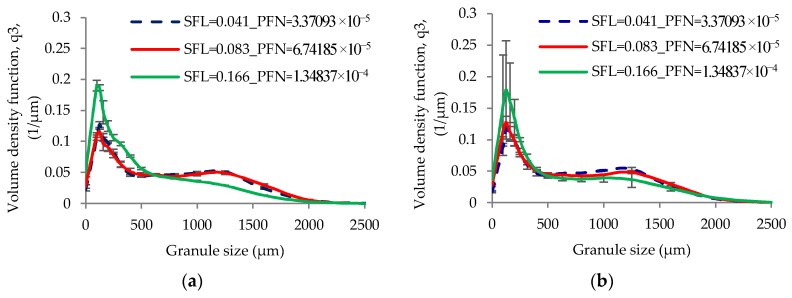

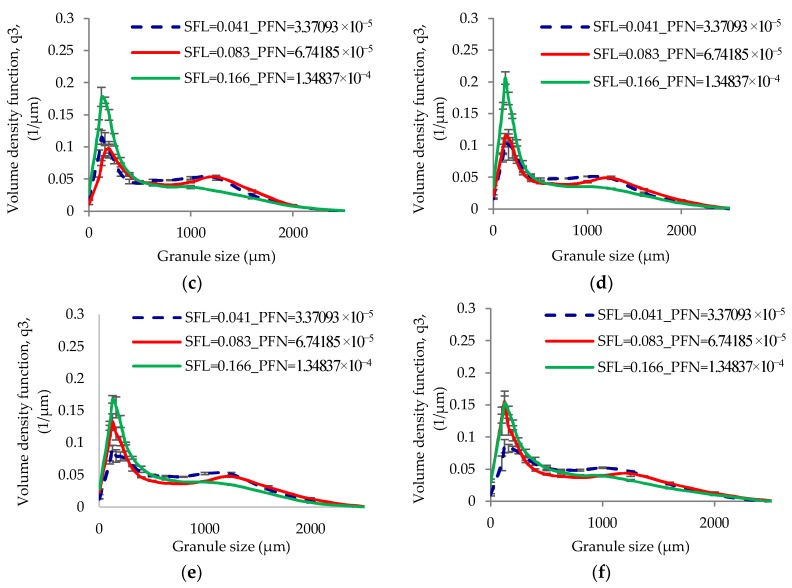
Granule size distribution of MCC (L/S 1) at different SFL–PFN at varying screw speed. (**a**) 150 rpm; (**b**) 250 rpm; (**c**) 400 rpm; (**d**) 500 rpm; (**e**) 750 rpm; and (**f**) 1000 rpm.

Figure 17 shows the size distribution of granules produced at varying SFL–PFN at L/S of 1.

#### 3.2.3. The Effect of Varying SFL–PFN at Different Screw Speed on Granule Shape

**Figure 18:**
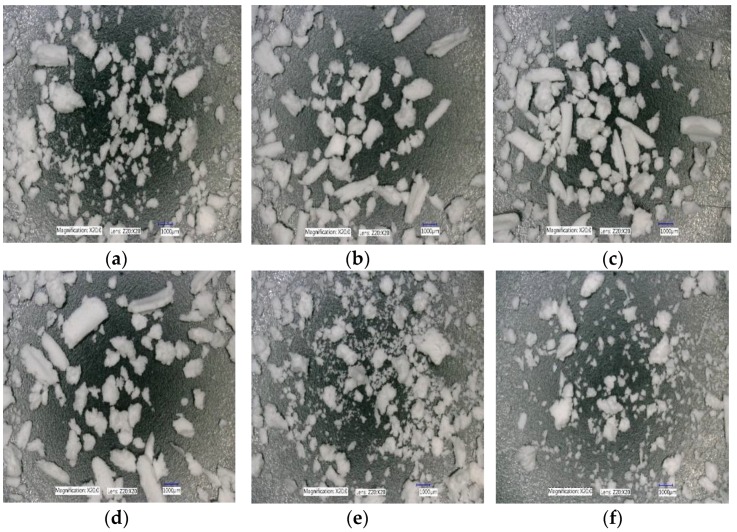
Microscopic images of MCC granules produced at varying screw speed at SFL—0.041 and PFN—3.37093 × 10^−5^. (**a**) 150 rpm; (**b**) 250 rpm; (**c**) 400 rpm; (**d**) 500 rpm; (**e**) 750 rpm; and (**f**) 1000 rpm.

**Figure 19:**
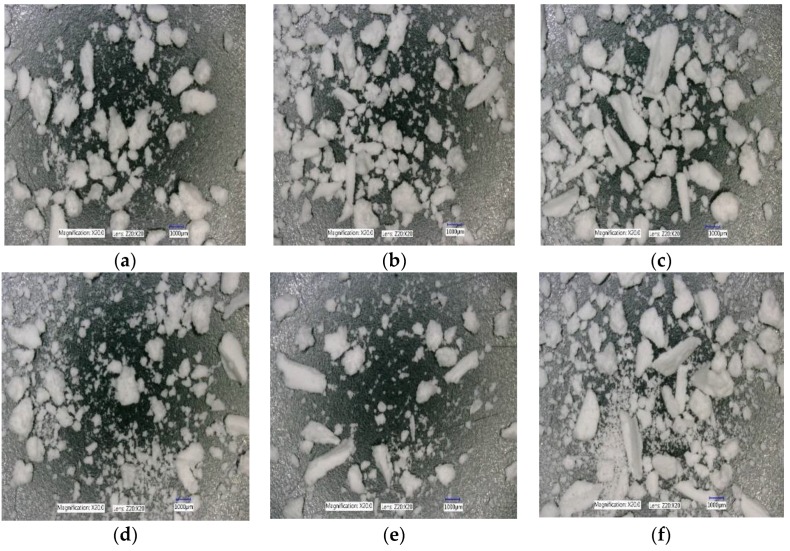
Microscopic images of MCC granules produced at varying screw speed at SFL—0.083 and PFN—6.74185 × 10^−5^. (**a**) 150 rpm; (**b**) 250 rpm; (**c**) 400 rpm; (**d**) 500 rpm; (**e**) 750 rpm; and (**f**) 1000 rpm.

**Figure 20:**
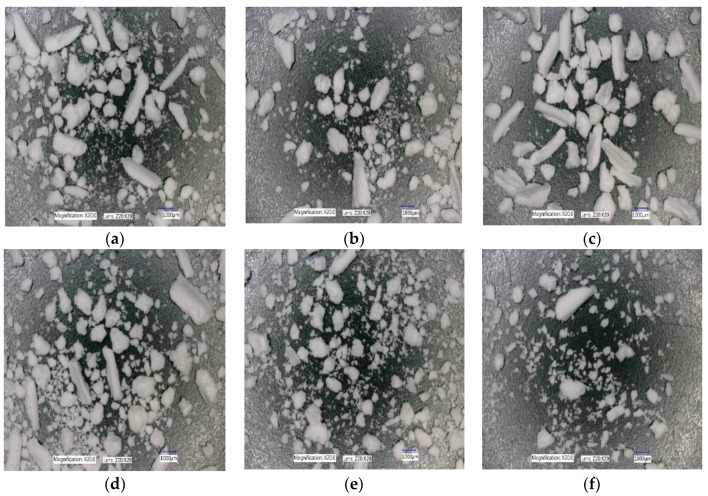
Microscopic images of MCC granules produced at varying screw speed at SFL—0.166 and PFN—1.34837 × 10^−4^. (**a**) 150 rpm; (**b**) 250 rpm; (**c**) 400 rpm; (**d**) 500 rpm; (**e**) 750 rpm; and (**f**) 1000 rpm.

The effect of varying SFL–PFN at different screw speed on granule shape is presented in Figure 18a–f, Figure 19a–f, and Figure 20a–f, respectively.

#### 3.2.4. The Effect of Varying SFL–PFN at Different Screw Speed on Tablet Tensile Strength

**Figure 21:**
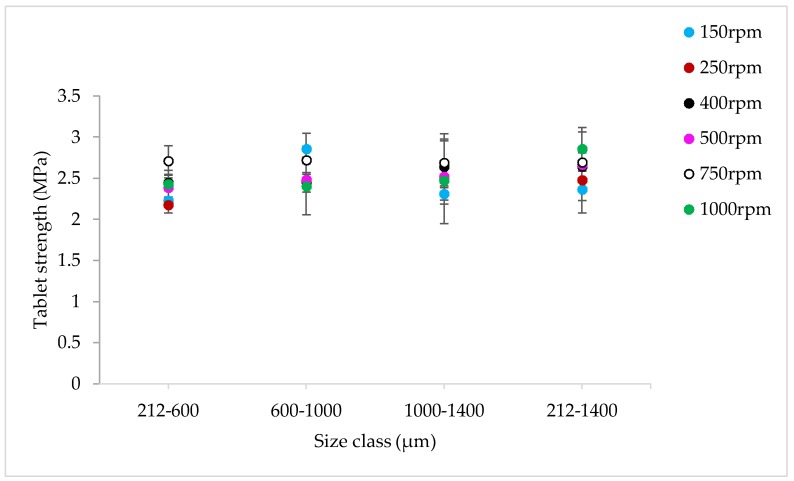
Tensile strength of tablets of MCC granules produced at varying screw speed at SFL—0.041 and PFN—3.37093 × 10^−5^ (L/S 1).

**Figure 22:**
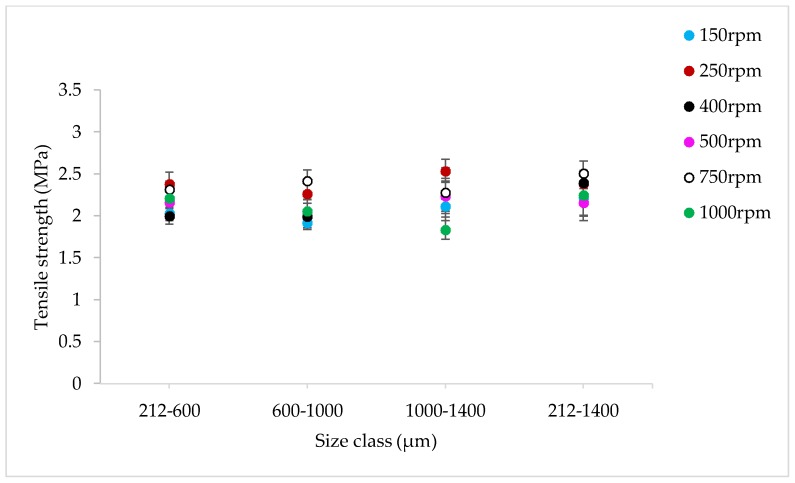
Tensile strength of tablets of MCC granules produced at varying screw speed at SFL—0.083 and PFN—6.74185 × 10^−5^ (L/S 1).

**Figure 23:**
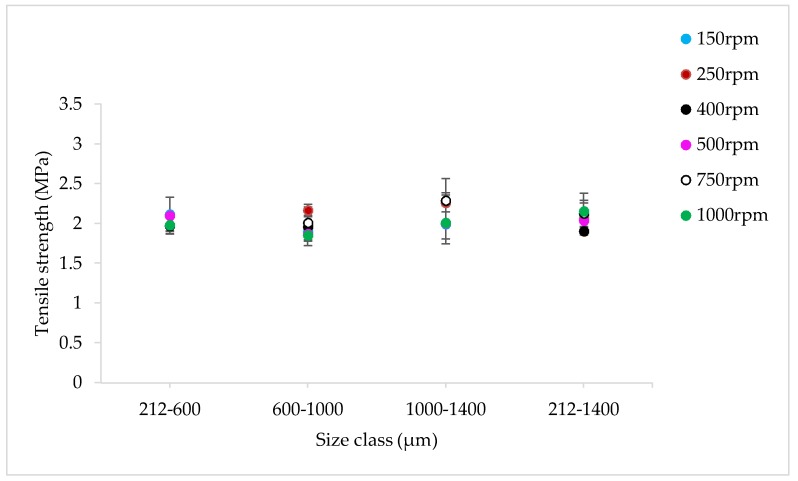
Tensile strength of tablets of MCC granules produced at varying screw speed at SFL—0.166 and PFN—1.34837 × 10^−4^ (L/S 1).

Figure 21, Figure 22 and Figure 23 show the tensile strength of tablets of MCC granules (with varying size ranges) produced at varying screw speed and SFL–PFN at L/S of 1.0.

## 4. Discussion

### 4.1. Lactose Powder

#### 4.1.1. The Effect of Varying SFL–PFN at Different Screw Speed and L/S on MRT

Figure 2 indicates that MRT decreases with the increase in screw speed at all fill levels (i.e., SFL–PFN). The reason for this is that increasing the screw speed increases the axial transport rate (drag flow rate) of the powder, thereby reducing the time the powder spends in the granulator. Comparing three SFL–PFN at various screw speeds, MRT was the highest at the lowest SFN–PFN (SFL—0.041 and PFN—1.71403 × 10^−5^) (i.e., low fill level). The reason for this is that the powder throughput or conveyance force [3], which pushes the powder in the forward direction in the granulator, was low at low fill levels at all screw speeds. In other words, at varying screw speed at a constant powder feed rate, the screws rotate while relatively starved of powder, and hence the fill level and throughput force mostly depends on the increase in the powder feed rate. In the present study, the powder feed rate was also varied in proportion to the increase in the screw speed to get the same specific feed load. Therefore, the relative barrel fill level, and thereby the throughput force, assumedly remains low for low SFL–PFN at all screw speeds. Thus, the throughput force at low SFN–PFN was relatively low and MRT was high when compared with those at higher SFL–PFN conditions, where the powder feed rate was higher for the corresponding screw speed.

As the fill level increased (i.e., SFL—0.083 and PFN—3.42806 × 10^−5^), MRT decreased sharply as a result of the increase in the throughput force. The further increase in the fill level (i.e., SFL—0.166 and PFN—6.85612 × 10^−5^) did not necessarily result in significant decline in MRT, especially at and after screw speed of 400 rpm, when compared with that at SFL—0.083 and PFN—3.42806 × 10^−5^. This indicates that there is a limit for MRT to change with the increase in SFL–PFN. As mentioned earlier, in the present study, the powder feed rate is varied in proportion to the screw speed in order to maintain the same specific feed load or fill level. At SFL—0.166 and PFN—6.85612 × 10^−5^, the powder feed rate was high, but so was the corresponding screw speed. Thus, screw speed could not control the fill level, and thereby throughput force and MRT, alone as the powder feed rate was also changing at same time.

A similar trend was noticed at an L/S of 0.1 (Figure 3), however, the magnitude of MRT was higher. This is expected since the powder was more wetted when compared with that at an L/S of 0.048, which led to the powder becoming more sluggish/cohesive or sticky, and thus flowing slower in the granulator. 

#### 4.1.2. The Effect of Varying SFL–PFN at Different Screw Speed and L/S on Granule Size

Figure 4 indicates that increasing SFN–PFN (i.e., fill level at varying screw speed) had no significant impact on the granule size. The median granule size ranged from 585 µm to 758 µm. Considering the difference in the mean residence time at varying fill levels at L/S 0.048, it was expected that granule size would also change in response to the screw speed and fill level. However, this was not the case, meaning that the difference in the mean residence time does not necessarily translate into the change in the granule size when the SFL is kept constant. The granule size can be more or less maintained at varying screw speed and fill level for simple placebo formulations, such as lactose in this case. The results can be explained by understanding the effect of screw speed on the wet granules’ flow within the twin screw granulator. At low screw speed, the peak shear rate, axial mixing, and the centrifugal force acting on the wet granules are low (despite keeping the same SFL at varying screw speeds). This means that the transport of wet granules in the granulator was mainly through a convective and less dispersive type of transport [28]. This means that the more dominant mechanism by which the granules are formed, broken, and/or reformed is the shearing of wet granules between the screw channel and barrel surface and in the intermeshing area between the screws. The sheared, broken, and surface wetted granules undergo a sequence of coalescence (granules attach to each other as they flow in close proximity at low screw speed) followed by breakage on the surface of the screw as it pushes the granules to opposite screws through the intermeshing area between them. 

As the screw speed increases, the peak shear rate, axial mixing, and the centrifugal force acting on the wet granules also increase. A high centrifugal force results in more dispersion transport and a stronger impact of wet granules on the barrel wall, the intermeshing section, and the other wet granules. Thus, the more dominant mechanism at high screw speed is the impact of wet granules. Some extent of shearing of wet granules in the intermeshing area between the screws also takes place at higher screw speed. 

Similar to low L/S (0.048), at L/S of 0.1 (Figure 5), granule size was maintained at varying fill level (SFN–PFN) at different screw speeds. However, the overall granule size increased and ranged from 829 µm to 1021 µm. This is expected because the amount of liquid available for the particles to attach was higher at L/S of 0.1. Comparing the results at varying fill levels at different L/S, it is clear that the amount of liquid added is a critical input variable to be considered while increasing the throughput of the twin screw granulator by increasing the SFL–PFN. 

The low L/S (0.048) produced granules with some degree of bimodality (i.e., mixed amounts of small and bigger granules) (Figure 6). The limited availability of granulation liquid at low L/S of 0.048 resulted in non-homogenous wetting of powder mass [11], meaning the formation of some liquid rich, bigger, stronger, and weaker granules and some poorly wetted, smaller, and weaker granules, which were relatively easy to break or attrite at shear and compressive forces within the granulator. This potentially resulted in skewed or bimodal granule size distribution [10,32]. As the L/S increased to 0.1, the liquid distribution in powder mass was relatively improved and the granules formed were more wetted and deformable [33] at the shear and compressive forces within the granulator. This resulted in the formation of granules with monomodal size distribution (i.e., less proportion of smaller granules) when compared with that at low L/S (Figure 7). 

#### 4.1.3. The Effect of Varying SFL–PFN at Different Screw Speed and L/S on Granule Shape

Figure 8 indicates that the granule shape (elongation) does not vary noticeably as the screw speed is increased at constant fill level (SFL–PFN) at low L/S of 0.048. In all cases, a mixed proportion of large (elongated) and small size granules were produced. The results agree with the findings from previous research, in which limited availability of granulation liquid produced mixed-shape granules [1,5,10]. At such liquid amounts (L/S 0.048), powder mass was not homogenously wetted, meaning some stronger, larger granules did not deform sufficiently and remained elongated as they rolled on the screw channel surface during their progression along the granulator barrel length [34]. These microscopic results support the size distribution of granules at varying fill level, where no significant difference in size was observed. 

Similar observations were noted for higher fill level of SFL—0.083, PFN—3.42806 × 10^−5^ and SFL—0.166, PFN—6.85612 × 10^−5^ (data not shown). This was expected since the L/S was maintained at all fill levels.

Microscopic images of granules produced at L/S of 0.1 at varying screw speed and SFL–PFN indicated the presence of relatively spherical granules when compared with L/S of 0.048 (data not shown). The shape of the granules did not vary noticeably when compared with different screw speed and SFL–PFN (data not shown). 

#### 4.1.4. The Effect of Varying SFL–PFN at Different Screw Speed and L/S on Tablet Tensile Strength

Figure 9, Figure 10, Figure 11, Figure 12, Figure 13 and Figure 14 indicate that tablet strength did not vary significantly at all conditions (i.e., varying SFL–PFN at two L/S). Interestingly, the granule size ranges from various conditions also did not affect the tablet tensile strength. The compression force used in the present study was same as in previous research on twin screw granulation [1,14], in which tablet strength did not vary significantly with respect to L/S or screw speed. It is likely that this compression force was high enough to overcome the strength of all granules in various ranges used in this study.

### 4.2. Microcrystalline Cellulose (MCC) Powder

#### 4.2.1. The Effect of Varying SFL–PFN at Different Screw Speed on MRT

Figure 15 indicates that mean residence time decreased with increasing screw speed at all three fill levels (SFN–PFN). Comparing three SFL–PFN values, MRT values drop significantly at high SFL (0.166)–PFN (1.34837 × 10^−4^). These results for MCC powder are similar in terms of trend to those for lactose powder (at L/S of 0.1) (see Figure 2 and Figure 3), where MRT decreased with increasing screw speed. However, the magnitude of MRT for MCC is clearly smaller than lactose. This can be described based on the difference in the PFN values and powder–liquid interaction for MCC and lactose. The PFN values for MCC are almost double the lactose PFN values (Table 1). The PFN values take into consideration the bulk density of the powder, which is higher in case of MCC than in lactose. Thus, the overall barrel fill level for MCC is higher when compared with that for lactose. Furthermore, unlike lactose, MCC granules do not become sticky (because of its insolubility in water) and flow better within granulator. Therefore, as a result of higher fill level and better flowability, MCC granules spent a relatively shorter time within the granulator, hence the lower MRT. 

#### 4.2.2. The Effect of Varying SFL–PFN at Different Screw Speed on Granule Size

Figure 16 shows that median granule size was similar at two fill levels [SFL (0.041 and 0.083) and PFN (3.37093 × 10^−5^ and 6.74185 × 10^−5^)] at varying screw speeds. As the fill level increased further (SFL—0.166, PFN—1.34837 × 10^−4^), the granule size remained similar at varying screw speed, but was smaller when compared with two other fill levels. There are two potential reasons for this effect: either very short residence time (as a result of high throughput force at high fill level) for the MCC powder to form sufficiently strong granules, or high shearing and friction between the granules–barrel wall and granules–granules because of a relatively high barrel fill level, which reduces the overall granule size. The MRT exhibited almost no impact on granule size in the case of lactose powder because of its solubility in water. In case of MCC (because of its insolubility in water), however, MRT showed its effect owing to a short duration for powder–liquid interactions to occur at high fill level and greater flowability of MCC powder within the granulator.

The size distribution results in Figure 17 support the median granule size data obtained at various SFL–PFN. At all three fill levels or SFL–PFN, the granule size distributions were bimodal at all screw speeds. High fill level (SFL—0.166, PFN—1.34837 × 10^−4^) resulted in more fines or small granules when compared with two other fill levels. As mentioned in case of median granule size, the production of small size granules or fines was potentially a result of reduced powder–liquid interaction because of a shorter residence time or high shearing.

#### 4.2.3. The Effect of Varying SFL–PFN at Different Screw Speed on Granule Shape

Figure 18, Figure 19 and Figure 20 show the microscopic images of granules produced at varying SFL–PFN. In all cases, the granules produced were elongated in shape and comprised of small and large granules. This supports the size distribution of granules at three different fill levels, where higher fill level (SFL—0.166, PFN—1.34837 × 10^−4^) indicated the presence of more fines when compared with lower fill levels. The high compressibility of MCC (plastically deforming powder) adds to the formation of some elongated, flaky granules during twin screw wet granulation [32,35].

#### 4.2.4. The Effect of Varying SFL–PFN at Different Screw Speed on Tablet Tensile Strength

The tensile strength of tablets does not change significantly at varying size or SFL–PFN (Figure 21, Figure 22 and Figure 23). In all cases, the tensile strength of tablets of MCC granules ranged between ~2 MPa to 3 MPa, which is clearly higher than that of tablets of lactose granules. It is known that MCC is a water-insoluble, plastically-deforming powder with high compressibility, while lactose is water-soluble and brittle in nature and has lower compressibility [35]. Hence, the produced granules of lactose may be relatively stronger than the MCC granules. The weaker MCC granules compressed better than the stronger lactose granules and produced stronger tablets.

Furthermore, the tablet tensile strength results for MCC are not fully aligned with the granule size results. The granule size at SFL of 0.041 and 0.083 was similar, while higher SFL of 0.166 resulted in relatively smaller granules. The tablet tensile strength for all three SFLs was similar. As mentioned earlier, this may be a result of high compression force during tableting, which overcomes the strength of granules in various size ranges. 

## 5. Conclusions

Through this study, it was shown that the specific feed load or powder feed number at a given L/S can act as a surrogate for the barrel fill level, which can be used to control and maintain the granule size and shape. The control on the tablet tensile strength at varying SFL–PFN was not conclusive as it did not change noticeably, even after increasing the liquid granulation amount. This means that the compression force may need further optimization (lower compression force) in order to see any possible effect of varying processing conditions on the tablet tensile strength. It was also found that the material property plays an important role in studying fill level. Lactose maintained the granule size at all fill levels because it dissolved in water and formed liquid bridges that helped to bind material together to form relatively stronger granules. On the contrary, MCC, which is insoluble in water, produced a larger proportion of smaller granules at a higher fill level, owing to the limited interaction between powder and liquid as a result of high throughput force and short residence time. Thus, it is concluded that along with fill level, material property and residence time are also important factors when considering increasing the throughput (scaling out) of twin screw granulators. There is scope to validate the approach of using SFL–PFN for increasing the machine throughput while keeping the granule and tablet quality attributes similar using a range of materials with varying physical and mechanical properties.

## Figures and Tables

**Table 1:** Experimental plan. SFL—specific feed load; PFN—powder feed number; MCC—microcrystalline cellulose.

Expt. No.	Powder Feed Rate (g/min)	Screw Speed (rpm)	SFL (g)	PFN (-) for Lactose	PFN (-) for MCC
1	6.25	150	0.041	1.71403 × 10^−5^	3.37093 × 10^−5^
2	10.41	250			
3	16.66	400			
4	20.83	500			
5	31.25	750			
6	41.66	1000			
7	12.50	150	0.083	3.42806 × 10^−5^	6.74185 × 10^−5^
8	20.83	250			
9	33.33	400			
10	41.66	500			
11	62.50	750			
12	83.33	1000			
13	25.00	150	0.166	6.85612 × 10^−5^	1.34837 × 10^−4^
14	41.66	250			
15	66.66	400			
16	83.33	500			
17	125.00	750			
18	166.66	1000			
